# Novel Paramyxovirus Associated with Severe Acute Febrile Disease, South Sudan and Uganda, 2012

**DOI:** 10.3201/eid2002.131620

**Published:** 2014-02

**Authors:** César G. Albariño, Michael Foltzer, Jonathan S. Towner, Lory A. Rowe, Shelley Campbell, Carlos M. Jaramillo, Brian H. Bird, DeeAnn M. Reeder, Megan E. Vodzak, Paul Rota, Maureen G. Metcalfe, Christina F. Spiropoulou, Barbara Knust, Joel P. Vincent, Michael A. Frace, Stuart T. Nichol, Pierre E. Rollin, Ute Ströher

**Affiliations:** Centers for Disease Control and Prevention, Atlanta, Georgia, USA (C.G. Albariño, J.S. Towner, L.A. Rowe, S. Campbell, B.H. Bird, P. Rota, M.G. Metcalfe, C.F. Spiropoulou, B. Knust, J.P. Vincent, M.A. Frace, S.T. Nichol, P.E. Rollin, U. Ströher);; Geisinger Medical Center, Danville, Pennsylvania, USA (M. Foltzer, C.M. Jaramillo);; Bucknell University, Lewisburg, Pennsylvania, USA (D.M. Reeder, M.E. Vodzak)

**Keywords:** Paramyxoviridae, metagenomics, zoonosis, diagnostics, rash, South Sudan, Uganda, viruses, Sosuga virus, bats, rubula-like virus

## Abstract

In 2012, a female wildlife biologist experienced fever, malaise, headache, generalized myalgia and arthralgia, neck stiffness, and a sore throat shortly after returning to the United States from a 6-week field expedition to South Sudan and Uganda. She was hospitalized, after which a maculopapular rash developed and became confluent. When the patient was discharged from the hospital on day 14, arthralgia and myalgia had improved, oropharynx ulcerations had healed, the rash had resolved without desquamation, and blood counts and hepatic enzyme levels were returning to reference levels. After several known suspect pathogens were ruled out as the cause of her illness, deep sequencing and metagenomics analysis revealed a novel paramyxovirus related to rubula-like viruses isolated from fruit bats.

Paramyxoviruses comprise a large family of viruses, including pathogens that cause severe disease in humans (*1*). Worldwide, >100 paramyxoviruses have been identified in bats and rodents (*2*–*4*). Among these, few have been shown to be pathogenic to humans, possibly because of limited host range and/or infrequent exposure. We describe a novel rubula-like virus that was associated with a severe acute febrile illness in a woman. The patient was a wildlife biologist who had participated in a 6-week field expedition to South Sudan and Uganda. During this expedition, she had been exposed to bats and rodents of >20 species while wearing different levels of personal protective equipment.

## Clinical Presentation

During the summer of 2012, a 25-year-old female wildlife biologist participated in a 6-week field expedition to South Sudan and Uganda, where she traveled to remote rural areas collecting bats and rodents for ecologic research. In the course of her duties, she manipulated animals in traps and mist nets, performed dissections, collected blood and tissues, and visited caves with large bat populations. She received no injuries from sharp objects and no bites or scratches from the animals with which she was working. She occasionally used respiratory protection when working with animals and specimens, and she wore a respirator while in caves. During her trip, she had no known contact with ill members of the field team, no contact with health care facilities, and no sexual contacts. She had been vaccinated against hepatitis A and B, yellow fever, measles, mumps, rubella, rabies, polio, tetanus/diphtheria, and typhoid fever, and she fully complied with a malaria prophylaxis regimen of atovaquone/proguanil. Her medical history included migraines and treatment of presumptive malaria with artemether/lumefantrine during a similar expedition the previous year.

Five days after returning to the United States, the woman was evaluated in the emergency department for a 2-day history of fever, malaise, headache, generalized myalgia and arthralgia, neck stiffness, a metallic taste, and sore throat. Results of rapid malaria test, performed on the day of fever onset, were negative. Other laboratory results and the patient’s vital signs at the time of admission are summarized in the Table. The patient seemed to be fatigued but alert and oriented; she was anicteric, and she had no nuchal rigidity or focal neurologic deficits. Mild erythema of the soft palate without ulcerations or exudates was noted. The spleen tip was palpable despite absence of adenopathy. 

**Table:** Vital signs and laboratory results for patient infected with a novel paramyxovirus related to rubula-like viruses isolated from fruit bats

DSO	DH	Max temp	Max pulse	SBP	WBC	Plate	Creat	AST	ALT	LDH,	TB	PT/INR ratio	TG	Ferr	qRT-PCR, C_t_†	IgM ELISA†	IgG ELISA†
2	1	40.1	90	112	1.62	115	0.8	93	19	687	0.2						
3	2	40.1	79	112	1.53	93	0.7	133	20		0.1	17.2/1.45			29.5	<50	<50
4	3	40.4	77	103	1.06	77	0.6	164	28		0.1	15.8/1.29	120				
5	4				1.02	65	0.7	319	134		0.1	13.6/1.06		17,840			
6	5	38.6	77	102	0.95	62	0.6	615	261		0.1	13.7/1.07	127	11,595			
7	6				1.46	79	0.6	589	298		0.2	14.4/1.14		7,309			
8	7				1.64	84	0.5	516	299		0.1	15.5/1.26		3,371			
9	8				0.96	123	0.5	342	259		0.1	16.9/1.41			36.3	>1,600	>1,600
10	9				1.20	154	0.4	170	186		0.1	17.4/1.47			36.9	>1,600	>1,600
11	10				2.19	222	0.4	185	188		0.1	14.7/1.18			Neg	>1,600	>1,600
12	11				2.61	220	0.4	107	149		0.2	13.2/1.02			Neg	>1,600	>1,600
13	12				5.62	335	0.4								Neg		
14	13				5.79	387	0.4								Neg		
15	14	36.8	67	98	4.71	437	0.5						212		Neg	>1,600	>1,600
30					3.71	221		19	15		0.3						
60					5.44	348								12.4	Neg	>400	>1,600

*Shading indicates values outside reference range; blank cells indicate data not obtained. DSO, days from symptom onset; DH, day of hospitalization; max temp, maximum temperature, °C; max pulse; maximum pulse rate, beats/minute; SBP, systolic blood pressure, mm Hg; WBC, leukocytes ,× 1,000/µL; plate, platelets × 1,000/µL; creat, creatinine, mg/dL; AST, aspartate aminotransferase, IU/L; ALT, alanine aminotransferase, IU/L; LDH, lactate dehydrogenase, IU/L; TB, total bilirubin, mg/dL; PT/INR, prothrombin time/international normalized ratio; TG, triglycerides, g/dL; ferr, ferritin, ng/mL; qRT-PCR, quantitative reverse transcription PCR; C_t_, cycle threshold; neg, negative.  †Assays were developed after the virus genome was determined.

Examination of heart, abdomen, and lungs (including chest radiographs) revealed no abnormalities. No rash or synovitis was noted. Treatment with intravenous ceftriaxone was begun for possible typhoid fever, and artemether/lumefantrine was continued for presumptive malaria.

**Figure 1:**
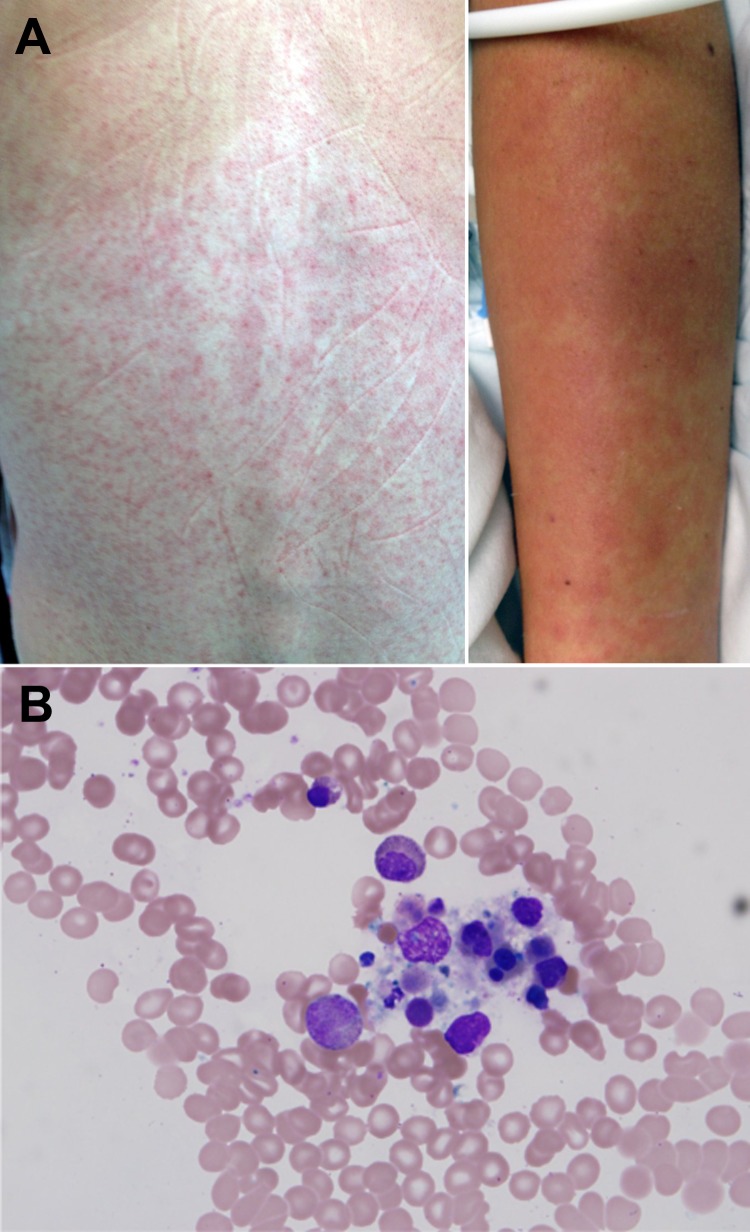
A) Maculopapular eruption observed on the back and arms of 25-year-old female wildlife biologist infected with a novel paramyxovirus related to rubula-like viruses isolated from fruit bats, on hospitalization day 2. B) Bone marrow biopsy sample showing macrocytic hemophagocytosis (possible granulocyte infiltration).

On hospital day 2, a maculopapular rash erupted over the patient’s trunk (Figure 1, panel A), several small aphthous ulcers appeared on her soft palate, and she had mild diarrhea. As long as the fever persisted, clear pulse/temperature dissociation was present (positive Faget sign); however, hemodynamics, oxygenation, and renal function were stable. Doxycycline was added for the expanded differential diagnosis of a rickettsial illness or plague. On hospital day 3, fever, headache, and myalgia persisted, and the patient experienced bloody emesis, mild diarrhea positive for occult blood but without frank hematochezia or melena, and minimal diffuse abdominal tenderness. Her menstrual period occurred without substantial menorrhagia. The rash became confluent; a centrifugal distribution and prominent petechia appeared at sites of trauma or pressure.

The possibility of hemophagocytosis was considered, and a bone marrow biopsy sample was obtained on day 4. The sample showed a mild increase in macrocytic hemophagocytosis and pancytopenia with a hypocellular marrow with myeloid hyperplasia and erythroid hypoplasia (Figure 1, panel B).

The fever slowly but progressively decreased, and the patient improved over the next few days; the last recorded fever was on hospital day 9. Abdominal pain and diarrhea resolved. Ceftriaxone was discontinued after 8 days. When the patient was discharged on hospital day 14, arthralgia and myalgia had improved, oropharynx ulcerations had healed, the rash had resolved without desquamation, and blood counts and hepatic enzyme levels were returning to reference limits. Considerable sequelae (myalgia, arthralgia, headache, malaise, and fatigue) persisted for several months.

## Diagnostic Workup

The initial suspected diagnosis was hematophagocytic syndrome (hemophagocytic lymphohistiocytosis). This clinical syndrome has been associated with a variety of viral, bacterial, fungal, and parasitic infections, as well as collagen–vascular diseases and malignancies. Initial diagnostic testing for various infectious diseases included blood screening for respiratory viruses, HIV, cytomegalovirus, and malaria parasites; all results were negative.

On hospital day 2, a diagnosis of a viral hemorrhagic fever was considered, and blood specimens were sent to the Centers for Disease Control and Prevention (CDC) for testing. Molecular testing results were negative for rickettsiae, filoviruses (Marburgviruses and Ebolaviruses), selected bunyaviruses (Rift Valley fever virus, Crimean Congo hemorrhagic fever virus), arenaviruses (Lassa, Lujo, and lymphocytic choriomeningitis viruses), and several arboviruses (yellow fever, dengue, O'nyong-nyong, chikungunya, and Zika viruses).

A pathogen-discovery deep-sequencing protocol was followed, as described (*5*,*6*). In brief, total RNA was extracted from blood and serum samples obtained 3 days after symptom onset; RNA was nonspecifically amplified with previously described primers (*7*). The cDNA library was sequenced on a 454 FLX Genome Sequencer (Roche Diagnostics, Indianapolis, IN, USA). Unassembled sequences were translated and compared with the nonredundant protein database from the National Center for Biotechnology Information by using a BLASTx algorithm (www.ncbi.nlm.nih.gov/blast/Blast.cgi). The sequence reads linked to the BLASTx results were distributed into taxa by using MEGAN (*8*). Metagenomic analysis revealed a novel paramyxovirus in the patient’s blood and serum; the virus was most closely related to Tuhoko virus 3, a rubula-like virus (family *Paramyxoviridae*) recently isolated from *Rousettus leschenaultii* fruit bats in southern China (*4*). The next-generation sequence reads with homology to Tuhoko virus 3 covered ≈25% of the expected complete virus genome. Based on the sequences obtained, a series of primers were designed to amplify overlapping fragments spanning the complete genome of this novel virus. A detailed list of primers is available upon request. Amplicons of different sizes were obtained by standard reverse transcription PCR (RT-PCR) and sequenced by the standard Sanger method (*5*,*6*).

**Figure 2:**
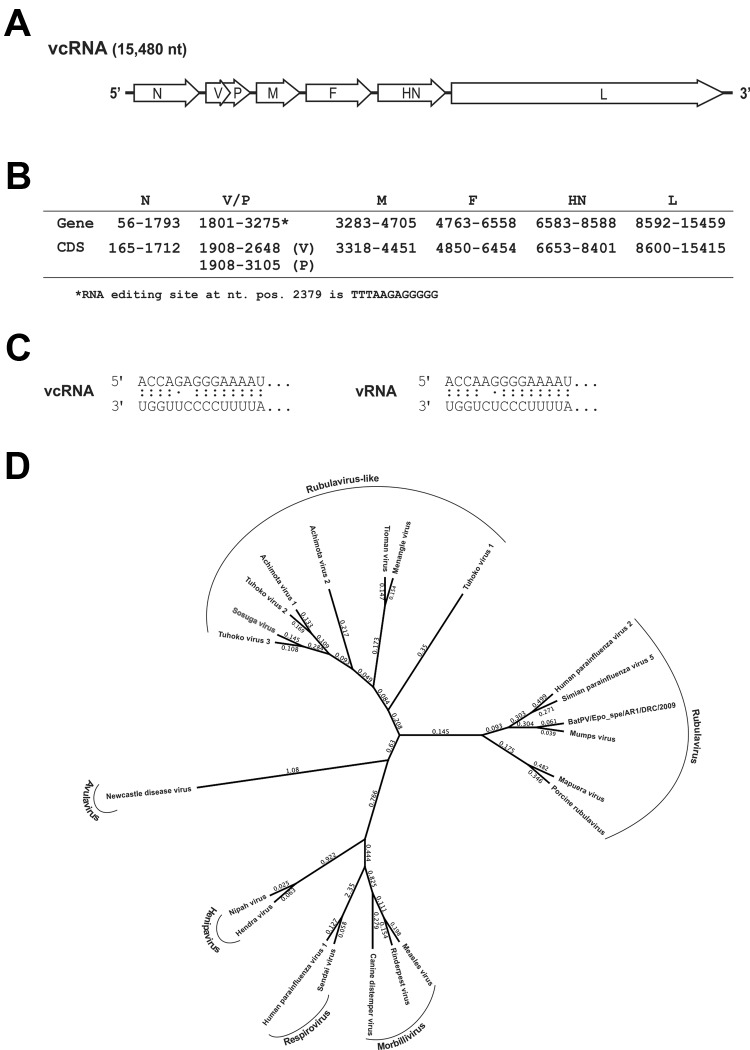
A) Organization of the viral genome of novel paramyxovirus related to rubula-like viruses isolated from fruit bats was determined from the full-length sequence. B) Localization of the predicted viral genes and open reading frames (ORFs). The V/P edition site is predicted from the similarity to Tuhoko virus 3. C) Terminal sequences were determined by standard rapid amplification of cDNA ends (RACE) methods. The complementarity of terminal sequences is shown in vRNA and vcRNA sense. D) Amino acid sequences of the nucleocapsid (N) protein of 22 representative paramyxovirus sequences were aligned by using the MUSCLE algorithm (CLC Genomics Workbench version 6.0.1; CLC bio, Cambridge, MA, USA). The phylogenetic analysis was conducted with a Bayesian algorithm (Mr.Bayes, Geneious version 6.1.5, www.geneious.com/). NP sequences were extracted from the complete genomic sequences in GenBank: KF774436 (Sosuga virus [SosV]), GU128082 (Tuhoko virus 3 ), GU128081 (Tuhoko virus 2), GU128080 (Tuhoko virus 1), AF298895 (Tioman virus), NC_007620 (Menangle virus), JX051319 (Achimota virus 1), JX051320 (Achimota virus 2), NC_003443 (human parainfluenza virus type 2), AF052755 (simian parainfluenza virus 5), HQ660095 (bat paramyxovirus Epo_spe/AR1/DRC/2009), NC_002200 (mumps virus), NC_009489 (Mapuera virus), NC_009640 (porcine rubulavirus), NC_001498 (measles virus), NC_006296 (rinderpest virus), NC_001921 (canine distemper virus), NC_001552 (Sendai virus), NC_003461 (human parainfluenza virus type 1), NC_002728 (Nipah virus), NC_001906 (Henra virus), NC_002617 (Newcastle disease virus). vcRNA, viral complementary RNA; N, nucleocapsid protein; V/P, V protein; M, matrix protein; F, fusion protein; HN, hemagglutinin-neuraminidase; L, molecular weight DNA ladder; CDS, coding sequence; nt pos. nucleotide position; vRNA, viral RNA.

This novel paramyxovirus is provisionally named Sosuga virus in recognition of its probable geographic origin (South Sudan, Uganda). The complete genome of Sosuga virus was 15,480 nt long and conformed to the paramyxovirus rule of 6 (*1*). The genome organization (Figure 2, panels A, B) resembled that of most paramyxoviruses, containing 6 genes, *N*, *V/P*, *M*, *F*, *HN*, and *L*, encoding the 7 viral proteins: nucleocapsid (N), V protein (V), phosphoprotein (P), matrix protein (M), fusion protein (F), hemagglutinin–neuraminidase (HN), and polymerase (L). The sequence of the RNA editing site in the *V/P* gene is identical to that of Tuhoko virus 3 (*4*). The faithful transcription of V/P generates the V mRNA, and a GG insertion at the editing site generates the P mRNA. In addition, the terminal 5′ and 3′ sequences of the virus were experimentally determined (Figure 2, panel C) by rapid amplification of cDNA ends as described (*9*).

Pairwise comparison of the full-length sequence of Sosuga virus with the closest related viruses showed 61.6%, 53.1%, and 51.4% identities, respectively, with Tuhoko virus 3, Achimota virus 1, and Achimota virus 2 (Achimota viruses were isolated from the *Eidolon helvum* fruit bat in Ghana) (*3*). When the deduced amino acid sequences of Sosuga virus were compared with those of Tuhoko virus, 3 proteins revealed overall amino acid identities ranging from 57.4% (HN) to 84% (N). Phylogenetic analysis of Sosuga virus and other paramyxoviruses clearly showed that Sosuga virus clusters with other bat-borne rubula-like viruses, which are closely related to rubulaviruses but have not yet been classified as such (Figure 2, panel D).

## Virus Isolation

**Figure 3:**
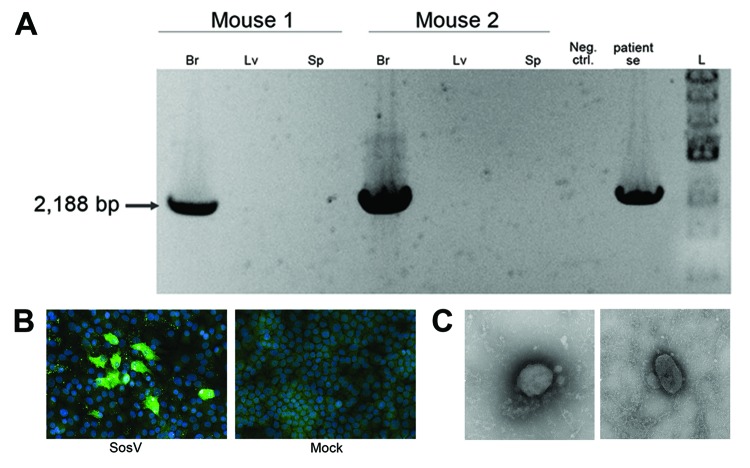
A) Virus isolation confirmed by reverse transcription PCR. SosV was isolated after intracranial and intraperitoneal inoculation into 2-day-old suckling mice. A specific reverse transcription PCR designed to amplify 2,188 bp of the SosV genome was performed by using RNA from brains (Br), liver (Lv), and spleen (Sp) of the euthanized animals. Viral RNA was found only in the brain, not in liver or spleen. B) Propagation of SosV in cell culture. Homogenized tissues (brain, liver, and spleen) were used to infect H292 cells. Fixed monolayers were stained with convalescent-phase serum from the patient and anti-human AlexaFluor 488 antibody (Invitrogen, Grand Island, NY, USA). C) Sosuga virus particle. Virus morphologic appearance was examined by taking supernatants from infected Vero-E6 cells, clarifying by slow-speed centrifugation, and depositing on grids for negative staining and examination by transmission electron microscopy. Pleomorphic virions can be observed. Neg.ctrl, negative control; Se, serum; SosV, Sosuga virus.

Virus isolation was attempted by inoculating monolayers of Vero-E6, Vero-SLAM, and H292 cells (mucoepidermoid carcinoma cells from human lungs) with patient blood and serum collected 3 days after symptom onset, but no virus isolate was obtained. As an alternative, 10 μL of the blood sample was also inoculated intracranially and intraperitoneally into 2-day-old suckling mice, which were then observed for 28 days for signs of illness. Neurologic signs developed 9–10 days after inoculation in 2 of the 20 mice; these 2 mice were euthanized 2 days later. To confirm the presence of the virus, we extracted total RNA from liver, spleen, and brains of the euthanized animals and used it as input in a specific RT-PCR designed to amplify a 2,188-bp fragment partially spanning the virus *HN* and *L* genes. Consistent with virus replication and observed neurologic signs, viral RNA was found in the brain but not in liver or spleen samples (Figure 3, panel A). 

Brain homogenates from the euthanized mice were inoculated into fresh monolayers of Vero-E6 cells and H292 cells; 12 days after infection, a cytopathic effect, with cell rounding but no syncytia formation, became evident. Virus antigen was detected by immunofluorescence in both cell lines by using patient’s convalescent-phase serum, collected 50 days after symptom onset (Figure 3, panel B). Moreover, transmission electron microscopy used to examine virus morphology showed pleomorphic virions, consistent with those of paramyxoviruses (Figure 3, panel C).

## Development of New Diagnostic Assays

Because the patient seemed to have acquired the infection during her African research expedition, where she had had extensive contact with rodents and bats, other persons who also come in contact with bats or rodents, such as field biologists, local residents, or ecotourists, might be at risk for infection. This potential public health threat prompted us to develop diagnostic assays for the rapid detection of Sosuga virus.

First, we developed a TaqMan real-time RT-PCR selective for the *N* gene and tested it on all available serum and blood samples from the patient. This test showed that the patient’s viremia peaked early in the course of the infection (cycle threshold 29.5 on day 3 after symptom onset), coinciding with the period of high fever and diverse irregularities in blood parameters (Table). By day 9, the viremia had decreased (cycle threshold 36.3); viremia was undetectable 11 days after symptom onset.

Second, we developed a new ELISA specific for Sosuga virus by using the virus recombinant nucleocapsid protein produced and purified from *Escherichia coli*. This assay was tested on all available serum samples from the patient (Table). Although IgG and IgM were not detectable on day 3 after symptom onset (titers <50), seroconversion (IgG and IgM titers >1,600) occurred 11 days after symptom onset. As expected, IgM levels later decreased (titer >400), and IgG levels remained high 50 days after symptom onset.

In addition, the new ELISA was tested for potential cross-reactivity with some common paramyxoviruses, including mumps and measles viruses. No cross-reactivity was detected on the ELISA plates when control serum from patients with high levels of IgG against mumps and measles viruses was used, a desired feature in a new diagnostic assay because most persons have IgG to these viruses as a result of vaccination or natural infection.

## Conclusions

A severe disease affected a wildlife biologist shortly after her return from rural Africa to the United States. Because of the disease characteristics (high fever and blood abnormalities) and travel history, a viral hemorrhagic fever was suspected, and clinical samples were rushed to CDC for investigation of a possible high-risk virus. After molecular and serologic diagnostic assays ruled out several well-known human pathogens (e.g., filoviruses, arenaviruses, phleboviruses, flaviviruses, and rickettsiae) as the cause of the patient’s illness, a next-generation sequence approach was followed to detect a possible new infectious agent.

The combination of next-generation sequencing and metagenomic analysis identified a novel paramyxovirus; the virus genome was completely characterized by use of standard sequencing techniques. The complete virus sequence clearly indicated a relationship with other rubula-like viruses isolated from bats. Moreover, the novel virus was isolated from acute-phase serum samples by infecting suckling mice and propagating the virus in cell culture. 

The specific molecular and serologic diagnostic assays that we developed will facilitate rapid identification of this novel infectious agent should new cases occur. We used these assays to retrospectively investigate all available clinical samples from the patient, and the results revealed periods of viremia and seroconversion.

Although the exact source of the patient’s infection remains unknown, the sequence similarity with bat-derived rubula-like viruses is highly suggestive. In recent years, a large number of diverse paramyxoviruses have been detected in bats (*2*,*10*), but only Nipah and Hendra viruses (genus *Henipavirus*) are known to cause severe disease in humans (*11*). An investigation to detect Sosuga virus in African bats is currently under way.
